# Genetic variation in *clusterin* and risk of dementia and ischemic vascular disease in the general population: cohort studies and meta-analyses of 362,338 individuals

**DOI:** 10.1186/s12916-018-1029-3

**Published:** 2018-03-14

**Authors:** Liv Tybjærg Nordestgaard, Anne Tybjærg-Hansen, Katrine Laura Rasmussen, Børge G. Nordestgaard, Ruth Frikke-Schmidt

**Affiliations:** 1 Department of Clinical Biochemistry, Rigshospitalet, Blegdamsvej 9, DK-2100 Copenhagen, Denmark; 2 The Copenhagen City Heart Study, Frederiksberg Hospital, Nordre Fasanvej 57, DK-2000 Frederiksberg, Denmark; 3 The Copenhagen General Population Study and Gentofte Hospital, Herlev Ringvej 75, DK-2730 Herlev, Denmark; 4 Copenhagen University Hospitals and Department of Clinical Medicine, Faculty of Health and Medical Sciences, University of Copenhagen, Copenhagen, Denmark; 5 The Department of Clinical Biochemistry, Herlev and Gentofte Hospital, Herlev Ringvej 75, DK-2730 Herlev, Denmark

**Keywords:** Clusterin, Apolipoprotein E, Alzheimer’s disease, Vascular dementia, Ischemic cerebrovascular disease, Ischemic heart disease, Cohort study, Meta-analysis

## Abstract

**Background:**

Clusterin, also known as apolipoprotein J (apoJ), is one of the most abundantly expressed apolipoproteins in the brain after apolipoprotein E (apoE). Like the ε4 allele of the apolipoprotein E gene (*APOE)*, the clusterin gene (*CLU*) is a risk locus for Alzheimer’s disease, and may play additional roles in atherosclerosis pathogenesis. We tested whether genetic variation in *CLU* was associated with either Alzheimer’s disease or atherosclerosis-related diseases.

**Methods:**

We studied individual data on 103,987 participants from the Copenhagen General Population Study (CGPS) and the Copenhagen City Heart Study (CCHS). We genotyped a common *CLU* variant (rs9331896) and two common *APOE* variants (rs7412 and rs429358), defining the *ε2*, *ε3*, and *ε4*, alleles in CGPS and CCHS. All individuals in the CGPS and CCHS cohorts were followed from study inclusion to occurrence of event, death, emigration, or until 10 November 2014, whichever came first. Summary consortia data on 258,351 individuals from the International Genomics of Alzheimer’s Project (IGAP) and the Coronary Artery Disease Genome-wide Replication and Meta-analysis plus the Coronary Artery Disease (C4D) Genetics and 1000-Genomes-based genome-wide association studies (CARDIoGRAMplusC4D) were used in meta-analyses.

**Results:**

In CGPS and CCHS, multifactorially adjusted hazard ratios for Alzheimer’s disease, all dementia, vascular dementia, ischemic cerebrovascular disease, and ischemic heart disease were 1.18 (1.07–1.30), 1.09 (1.02–1.17), 0.96 (0.80–1.17), 1.02 (0.97–1.07), and 0.97 (0.93–1.01) per T allele, respectively. Multifactorially adjusted hazard ratios for Alzheimer’s disease and all dementia were 2.72 (2.45–3.01) and 2.21 (2.05–2.38) for the *APOE* ɛ4 allele. There was no interaction between rs9331896 in *CLU* and rs429358 (defining the ɛ4 allele) in *APOE* in predicting Alzheimer’s disease or all dementia (*P* = 0.39 and *P* = 0.21). In a meta-analysis including consortium data, the overall fixed- and random-effects odds ratios for Alzheimer’s disease per T allele were 1.16 (1.13–1.18) (*I*^*2*^ = 0.0%; *P* for heterogeneity = 0.89).

**Conclusions:**

A common variant in *CLU* was associated with a high risk of Alzheimer’s disease and all dementia in the general population but not with vascular dementia or ischemic vascular disease. Important novel aspects compared to previous studies are the incorporation of individual risk factor data, the exact causative ε4 allele, and several subtypes of dementia and atherosclerosis-related endpoints.

**Electronic supplementary material:**

The online version of this article (10.1186/s12916-018-1029-3) contains supplementary material, which is available to authorized users.

## Background

Genome-wide association studies (GWASa) have identified a number of risk variants for late-onset Alzheimer’s disease of which several share features of both β-amyloid and lipid handling in the brain [1, 2]. Among these, the most well known is the ε4 allele of apolipoprotein E (apoE) [3]. Recently, the gene encoding clusterin (*CLU*) has consistently been associated with risk of Alzheimer’s disease [2, 4–6]. Like apoE, clusterin may also play a role in peripheral lipid metabolism [7]. Whether genetic variation in *CLU* is associated with Alzheimer’s disease, lipid metabolism, or atherosclerosis-related diseases in the general population and whether these associations are independent of the *APOE* genotype remains unexplored.

In the brain, clusterin (also known as apoJ) is one of the most abundantly expressed apolipoproteins, second only to apoE [7]. These apolipoprotein particles are synthesized in astrocytes, are pivotal for brain cholesterol metabolism, and are attached to a high-density lipoprotein (HDL)-like particle [7, 8]. The clusterin molecule is a versatile chaperone that has the ability to bind to a wide array of physiological ligands putatively involved in Alzheimer’s disease pathology [9]. One of these is β-amyloid, for which clusterin mediates clearance from the brain through the blood–brain barrier to the peripheral circulation via megalin [9, 10]. In the peripheral circulation, clusterin is attached to HDL-particles [11] and has been observed in atherosclerotic lesions [12, 13], where clusterin may prevent low-density lipoprotein (LDL) oxidation by arterial wall cells [14]. Since vascular dementia, ischemic cerebrovascular disease, and ischemic heart disease are all characterized by atherosclerosis [15, 16], variation in *CLU* may play a role for these atherosclerosis-related diseases in addition to the well-established association with Alzheimer’s disease.

We tested the hypothesis that genetic variation in *CLU* is associated with risk of Alzheimer’s disease, all dementia, vascular dementia, ischemic cerebrovascular disease, and ischemic heart disease in the general population. Thus, we genotyped 103,987 individuals from the Danish general population for a common *CLU* variant identified in a GWAS [2] and for well-known risk variants in *APOE*, serving as a positive control for the trustworthiness of our studies. We further included consortia data from the International Genomics of Alzheimer’s Project (IGAP) including 74,046 individuals and from the Coronary Artery Disease Genome-wide Replication and Meta-analysis plus the Coronary Artery Disease (C4D) Genetics and 1000-Genomes-based GWAS (CARDIoGRAMplusC4D) including 184,305 individuals.

## Methods

The Copenhagen studies were approved by institutional review boards and Danish ethical committees, and were conducted according to the Declaration of Helsinki. Written informed consent was obtained from participants. All Copenhagen participants were white and of Danish descent. There was no overlap between studies.

### Participants

#### The Copenhagen General Population Study

The Copenhagen General Population Study (CGPS) is a study of the general population that was initiated in 2003 with ongoing enrollment [17–20]. Data were obtained from a questionnaire, a physical examination, and from blood samples. We included 93,833 participants from this study in the analysis. Participants with events before study entry were excluded for each specific endpoint.

#### The Copenhagen City Heart Study

The Copenhagen City Heart Study (CCHS) is a study of the general population that was initiated in 1976–1978 with follow-up examinations in 1981–1983, 1991–1994, and 2001–2003. Data were obtained from a questionnaire, a physical examination, and from blood samples. We included 10,154 participants from the 1991–1994 and 2001–2003 examinations of this study in the analysis. Participants with events before study entry were excluded for each specific endpoint.

#### International Genomics of Alzheimer’s Project

IGAP is a large two-stage study based upon GWASs of individuals of European ancestry. In stage 1, IGAP used genotyped and imputed data on 7,055,881 single-nucleotide polymorphisms (SNPs) to meta-analyze four previously published GWAS datasets consisting of 17,008 Alzheimer’s disease cases and 37,154 controls. These datasets were from the European Alzheimer’s Disease Initiative (EADI), the Alzheimer’s Disease Genetics Consortium (ADGC), the Cohorts for Heart and Aging Research in Genomic Epidemiology Consortium (CHARGE), and the Genetic and Environmental Risk in Alzheimer’s Disease Consortium (GERAD). In stage 2, 11,632 SNPs were genotyped and tested for association in an independent set of 8,572 Alzheimer’s disease cases and 11,312 controls. Finally, a meta-analysis was performed combining results from stages 1 and 2.

#### The Coronary Artery Disease Genome-wide Replication and Meta-analysis plus the Coronary Artery Disease (C4D) Genetics and 1000-Genomes-based GWAS (CARDIoGRAMplusC4D)

CARDIoGRAMplusC4D is a meta-analysis of GWASs, mainly of people of European descent using the 1000 Genomes phase 1 v3 training set with 38 million variants for imputing. The study investigated 9.4 million variants and involved 60,801 ischemic heart disease cases and 123,504 controls from 48 studies [21].

### Events

Information on diagnoses of Alzheimer’s disease, all dementia, vascular dementia, ischemic cerebrovascular disease, and ischemic heart disease was collected from the national Danish Patient Registry and the national Danish Causes of Death Registry. The national Danish Patient Registry has information on all patient contacts with all clinical hospital departments in Denmark since 1977, including emergency wards and outpatient clinics from 1995. The national Danish Causes of Death Registry contains data on causes of all deaths in Denmark, as reported by hospitals and general practitioners since 1977. In the World Health Organization’s (WHO) International Classification of Diseases (ICD), Alzheimer’s disease is ICD8 290 and ICD10 F00 and G30. The validity of Alzheimer’s disease ICD codes was ensured by the presence of the well-known association with ε4 in the present cohort [18], with risk estimates similar to those reported elsewhere [3, 22]. Vascular dementia is ICD10 F01. All dementia includes Alzheimer’s disease, vascular dementia, and unspecified dementia (ICD8 290.18 and ICD10 F03). The quality of these registry-based diagnoses has previously been validated [23]. For ischemic cerebrovascular disease (ICD8 433-435 and ICD10 I63, I64, G45), validation of ICD codes has been described previously [17]. In brief, information on diagnoses of ischemic cerebrovascular disease (transitory ischemic attacks, amaurosis fugax, and ischemic stroke) was collected by reviewing all hospital admissions and diagnoses entered in the national Danish Patient Registry and the national Danish Causes of Death Registry. Possible cerebrovascular events (hospitalized as well as non-hospitalized) were validated by trained physicians using the WHO definition of cerebrovascular disease. Diagnoses of ischemic heart disease (ICD 8410-414 and ICD10 I20-I25) were collected and verified by reviewing all hospital admissions and diagnoses entered in the national Danish Patient Registry, all causes of death entered in the national Danish Causes of death Registry, and medical records from hospitals and general practitioners. Ischemic heart disease was fatal or nonfatal myocardial infarction or characteristic symptoms of angina pectoris, including revascularization procedures [24].

The follow-up period began at the time of blood sampling (2003 and onward for CGPS and 1991–1994 or 2001–2003 for CCHS). Follow-up ended at occurrence of an event, death, or emigration, or on 14 November 2014 (last update of the registry), whichever came first. Median follow-up times were 6 years (range 0–22 years) for Alzheimer’s disease, all dementia, and vascular dementia, 6 years (range 0–23 years) for ischemic cerebrovascular disease, and 5 years (range 0–22) for ischemic heart disease. No individuals were lost to follow-up.

### Genotyping

An ABI PRISM 7900HT Sequence Detection System (Applied Biosystems Inc., Foster City, CA, USA) and Taqman-based assays were used to genotype for rs9331896 and *APOE* (rs7412 and rs429358). rs7412 and rs429358 defines the six common *APOE* genotypes (ε22, ε32, ε42, ε33, ε43, and ε44), as previously described [18].

### Laboratory analyses

Plasma total cholesterol, HDL cholesterol, triglycerides, apolipoprotein B, and apolipoprotein AI were measured using standard hospital assays (Boehringer Mannheim GmbH, Mannheim, Germany and Konelab, Thermo Fischer Scientific, Waltheim, MA, USA). LDL cholesterol was calculated with the Friedewald equation [25] when plasma triglycerides were ≤4.0 mmol/L (≤352 mg/dL) and otherwise measured directly (Konelab). All assays were assessed daily for precision using internal controls and four to 12 times yearly for accuracy with a Scandinavian external quality control program. ApoE was measured by nephelometry as previously described [18] with a BNII autoanalyzer using goat anti-human apoE polyclonal antibodies (OQDLG09, Dade Behring, Deerfield, Illinois, USA) or by turbidimetry with a Kone autoanalyzer (Konelab) using rabbit anti-human apoE polyclonal antibodies (A0077, Dako, Glostrup, Denmark). A human serum apoE calibrator (Apolipoprotein Standard Serum, OUPGG07, Siemens Healthcare Diagnostics, Ballerup, Denmark) was used for both nephelometry and turbidimetry.

### Other covariates

Body mass index is the measured weight in kilograms divided by the measured height in meters squared. Hypertension was defined as systolic blood pressure ≥140 mmHg, diastolic blood pressure ≥90 mmHg, and/or use of anti-hypertensive medication. Diabetes mellitus was defined as a self-reported disease, use of insulin or oral hypoglycemic agents, and/or non-fasting plasma glucose levels of more than 11 mmol/L (>198 mg/dL). Smoking, alcohol consumption, physical inactivity, education, use of lipid-lowering therapy as well as postmenopausal status and hormonal replacement therapy in women were all self-reported and dichotomized. Smoking was current smoking. High alcohol consumption was >14/21 units of alcohol per week for women/men (1 unit alcohol ~12 g). Physical inactivity was ≤4 h of light physical exercise in leisure time weekly. Low educational level was less than eight years in school.

### Statistical analysis

Data were analyzed using Stata/SE version 14.0 (Stata Corp., College Station TX, USA). *P* values < 0.001 are given as powers of 10. The Kruskal–Wallis test, Mann–Whitney U test, and Pearson’s χ2-test were used to evaluate continuous and categorical variables by genotype and disease status. Power was calculated using the “stpower logrank” command in Stata.

Cumulative incidences of Alzheimer’s disease and all dementia were plotted against age and genotype, using the method of Fine and Gray [17, 26], to account for the possibility of death as a competing event. *P* values were calculated using the “stcrreg” command in Stata. Similar cause-specific (censoring at death) Cox proportional hazards regression models with age as time scale and left truncation (delayed entry) were used to estimate hazard ratios for Alzheimer’s disease, all dementia, vascular dementia, ischemic cerebrovascular disease, and ischemic heart disease per rs9331896 T allele or as a function of the rs9331896 genotype. Age was accounted for using age as the time scale, which implies that age is automatically adjusted for. For Cox regression models, the proportionality of hazards over time was assessed by plotting -ln(−ln[survival]) versus ln(analysis time). There was no suspicion of non-proportionality. Cox regression models were multifactorially adjusted for age (as time scale), sex, body mass index, hypertension, diabetes mellitus, smoking, alcohol intake, physical inactivity, postmenopausal status and hormonal replacement therapy in women, lipid-lowering therapy, and educational level. Missing values (0.7%) were imputed (continuous covariates) or assigned a dummy value (categorical covariates). However, if only individuals with complete data were included, the results were similar to those reported. The interaction between rs9331896 and rs429358 (defining the ɛ4 allele) relating to risk of Alzheimer’s disease, all dementia, vascular dementia, ischemic cerebrovascular disease, and ischemic heart disease was evaluated by the inclusion of two-factor interaction terms in the Cox regression model, using a likelihood ratio test between models excluding and including the interaction term. To exclude that our results were related to the *APOE* genotype, adjustment for the *APOE* genotype as well as a restricted analysis for *APOE* ε33 individuals only were performed. Odds ratios in meta-analyses were calculated based on beta coefficients from CCHS, CGPS, IGAP, and the CARDIoGRAMplusC4D Consortium using the “metan” command in Stata.

## Results

Baseline characteristics of the 103,987 study participants by genotype are shown in Table 1. All risk factors were equally distributed among genotypes or dementia status (Table 1, Additional file 1). rs9331896 genotype frequencies were 37.1%, 47.4%, and 15.5% for rs9331896 TT, TC, and CC, respectively, and did not deviate from the Hardy–Weinberg expectation (*P* = 0.99).

**Table 1:** Characteristics of study participants by genotype

	CC	TC	TT	*P*
Number of individuals (%)	16,076 (15.5)	49,315 (47.4)	38,596 (37.1)	
Age (years)	58 (48–68)	58 (48–67)	58 (48–67)	0.4
Female (%)	55	55	55	0.9
Total cholesterol (mmol/L)	5.6 (4.9–6.4)	5.6 (4.9–6.3)	5.6 (4.9–6.4)	0.1
LDL cholesterol (mmol/L)	3.2 (2.6–3.9)	3.2 (2.6–3.9)	3.2 (2.6–3.9)	0.1
HDL cholesterol (mmol/L)	1.6 (1.2–1.9)	1.6 (1.2–1.9)	1.6 (1.2–1.9)	0.8
Triglycerides (mmol/L)	1.4 (1.0–2.1)	1.4 (1.0–2.1)	1.4 (1.0–2.1)	0.5
Body mass index (kg/m^2^)	26 (23–28)	26 (23–28)	26 (23–28)	0.3
Hypertension (%)	59	59	60	0.4
Diabetes mellitus (%)	4	4	4	0.3
Smoking (%)	21	21	21	1
Alcohol consumption (%)	17	17	18	0.6
Physical inactivity (%)	54	54	54	0.9
Postmenopausal (%)^a^	68	67	66	0.4
Hormonal replacement therapy (%)^a^	11	11	10	0.1
Lipid-lowering therapy (%)	10	11	11	0.02
Education < 8 years (%)	13	12	13	0.4

Values are median (interquartile range) or percentage, and are from the day of enrollment in 2003 and onwards for CGPS and 1991–1994 or 2001–2003 for CCHS. Hypertension was use of anti-hypertensive medication and/or a systolic blood pressure of 140 mmHg or greater, and/or a diastolic blood pressure of 90 mmHg or greater. Diabetes mellitus was self-reported disease, use of insulin, or oral hypoglycemic agents, and/or non-fasting plasma glucose levels of more than 11 mmol/L (>198 mg/dL). Smoking was current smoking. Alcohol consumption was >14/21 units per week for women/men (1 unit = 12 g alcohol, equivalent to one glass of wine or one beer (33 cL)). Physical inactivity was ≤4 hours per week of light physical activity in leisure time. Women reported menopausal status and use of hormonal replacement therapy. Lipid-lowering therapy was primarily statins (yes/no) and education was < 8 years of education

*HDL* high-density lipoprotein, *LDL* low-density lipoprotein

^a^For women only

### rs9331896 and risk of Alzheimer’s disease, all dementia, vascular dementia, ischemic cerebrovascular disease, and ischemic heart disease

**Fig. 1:**
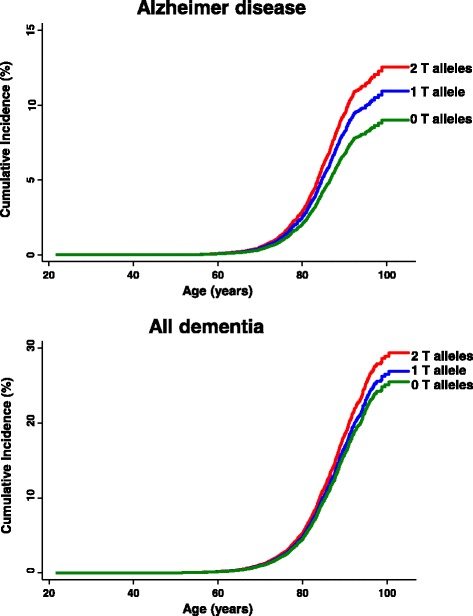
Cumulative incidences of Alzheimer’s disease and all dementia as a function of age and rs9331896. Fine–Gray models allowing for death as a competing event were used

**Fig. 2:**
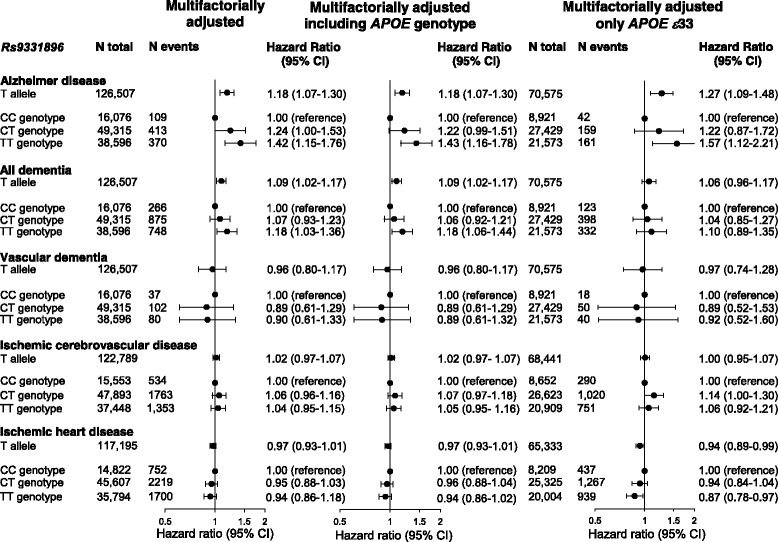
Risk of dementia and ischemic vascular disease as a function of rs9331896. Hazard ratios were multifactorially adjusted for age, sex, body mass index, hypertension, diabetes mellitus, smoking, alcohol consumption, physical inactivity, menopausal status and hormonal replacement therapy (only women), lipid-lowering therapy, and education (left panel). Hazard ratios were further adjusted for *APOE* genotype (middle panel). Analyses for Alzheimer’s disease, all dementia, and vascular dementia included 103,987 individuals. Analyses for ischemic cerebrovascular disease included 100,894 individuals and analysis for ischemic heart disease included 96,223 individuals. Analysis of individuals with the *APOE* ε33 genotype only included 57,923 individuals for Alzheimer’s disease, all dementia, and vascular dementia, 56,184 for ischemic cerebrovascular disease, and 53,538 for ischemic heart disease (right panel)

In CCHS and CGPS, the cumulative incidence of Alzheimer’s disease and all dementia increased stepwise from zero to two T alleles of rs9331896 (trend test *P* = 0.001 and *P* = 0.009) (Fig. 1), whereas the cumulative incidence of vascular dementia, ischemic cerebrovascular disease, and ischemic heart disease did not differ as a function of an increasing number of rs9331896 T alleles (Additional file 2). In CGPS and CCHS, multifactorially adjusted hazard ratios for Alzheimer’s disease, all dementia, and vascular dementia were 1.18 (95% confidence interval 1.07–1.30), 1.09 (1.02–1.17) and 0.96 (0.80–1.17) per T allele, respectively. For ischemic cerebrovascular disease and ischemic heart disease, the hazard ratios were 1.02 (0.97–1.07) and 0.97 (0.93–1.01) per T allele. No interactions between rs9331896 and rs429358 (defining the ε4 allele) relating to risk of Alzheimer’s disease, all dementia, vascular dementia, ischemic cerebrovascular disease, or ischemic heart disease were observed (*P* = 0.39 for Alzheimer’s disease, *P* = 0.21 for all dementia, *P* = 0.81 for vascular dementia, *P* = 0.06 for ischemic cerebrovascular disease, and *P* = 0.71 for ischemic heart disease). Multifactorially adjusted hazard ratios were 1.24 (1.00–1.53) for the rs9331896 TC versus CC and 1.42 (1.15–1.76) for TT versus CC genotypes for Alzheimer’s disease. Corresponding hazard ratios for all dementia were 1.07 (0.93–1.23) for the TC versus CC and 1.18 (1.03–1.36) for TT versus CC genotypes. These associations remained after adjustment for the *APOE* genotype, as well as in an analysis restricted to ε33 carriers for Alzheimer’s disease. No associations between rs9331896 and vascular dementia or ischemic cerebrovascular disease were observed (Fig. 2). A trend toward reduced risk was observed for ischemic heart disease, most evident when the analysis was restricted to ε33 carriers (*P* = 0.01). Similar estimates were observed when CGPS and CCHS were analyzed separately (Additional files 3 and 4).

We had 80% statistical power to detect hazard ratios above 1.28 for Alzheimer’s disease, 1.18 for all dementia, 1.49 for vascular dementia, 1.11 for ischemic cerebrovascular disease, and 1.10 for ischemic heart disease for TC heterozygotes. Similar estimates for TT homozygotes were 1.29 for Alzheimer’s disease, 1.19 for all dementia, 1.51 for vascular dementia, 1.12 for ischemic cerebrovascular disease, and 1.11 for ischemic heart disease.

### Comparison of risk estimates and frequencies for CLU rs9331896 and APOE rs425358 (ɛ4 allele)

**Fig. 3:**
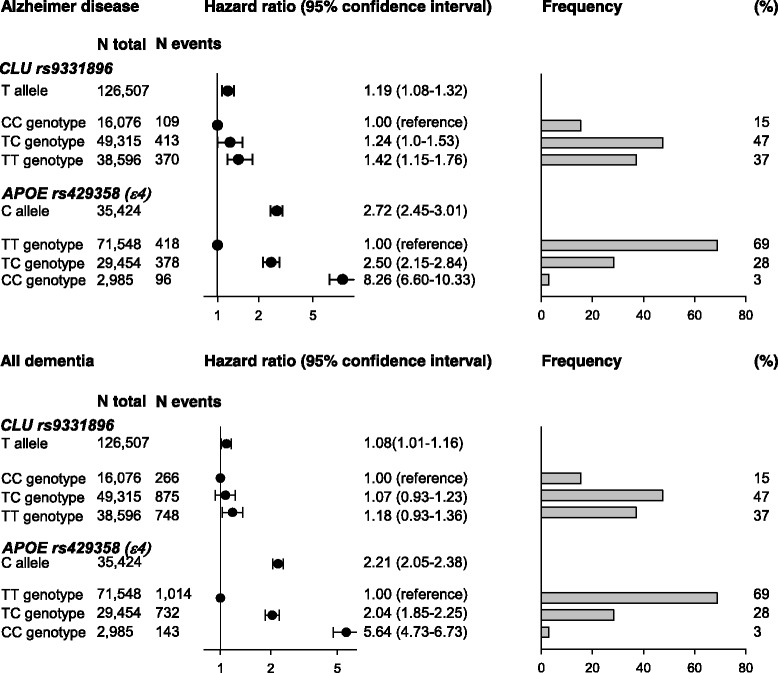
*Comparison of risk estimates and frequencies for CLU rs9331896 and APOE rs425358 (ɛ4 allele).* The *CLU* rs9331896 and *APOE* rs425358 (defining the ε4 allele) per allele effects are shown for comparison, as well as their frequencies

Results for rs9331896 are described above. Multifactorially adjusted hazard ratios for Alzheimer’s disease and all dementia were 2.72 (2.45–3.01) and 2.21 (2.05–2.38) per *APOE* rs425358 C allele (ɛ4 allele). Frequencies were 15%, 47%, and 37% for the rs9331896 CC, TC, and TT genotypes, respectively. Frequencies were 69%, 28%, and 3% for the *APOE* TT, CT, and CC genotypes, respectively (Fig. 3).

### Meta-analyses including consortia data

**Fig. 4:**
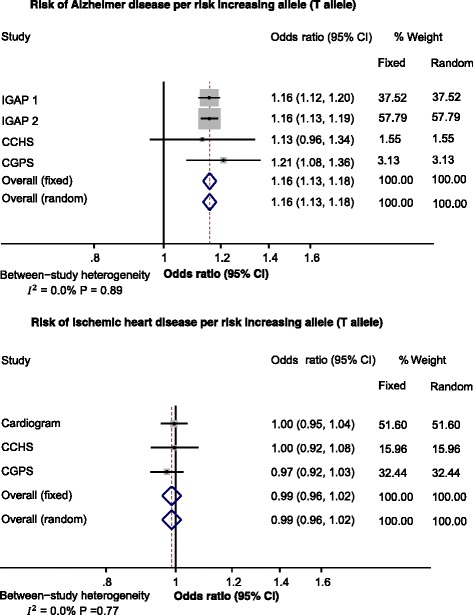
Meta-analyses of Alzheimer’s disease and ischemic heart disease risk per rs9331896 risk-increasing allele (T allele). Horizontal lines correspond to 95% confidence intervals by forest plots. Diamonds and broken vertical lines represent summary estimates. The confidence interval for the summary estimate corresponds to the width of the diamond. Gray shaded areas correspond to the weight of the study in the meta-analysis from the fixed effects model (right column). Cardiogram Coronary Artery Disease Genome-wide Replication and Meta-analysis plus the Coronary Artery Disease Genetics and 1000-Genomes-based GWAS, CCHS Copenhagen City Heart Study, CGPS Copenhagen General Population Study, CI confidence interval, IGAP International Genomics of Alzheimer’s Project

Meta-analyses on risk of Alzheimer’s disease as a function of risk-increasing T alleles in *CLU* included four independent studies. The overall fixed- and random-effects odds ratios were 1.16 (1.13–1.18) per risk-increasing allele (T allele) (*I*^*2*^ = 0.0%; *P* for heterogeneity = 0.89) (Fig. 4). Meta-analyses on risk of ischemic heart disease as a function of risk-increasing T alleles in *CLU* included three independent studies. The overall fixed- and random-effects odds ratios were 0.99 (95% confidence interval 0.96–1.02) (*I*^*2*^ = 0.0%; *P* for heterogeneity = 0.77) (Fig. 4).

### Genotype and plasma levels of lipids, lipoproteins, and apolipoproteins

The rs9331896 variant was associated with slightly higher plasma levels of apolipoprotein B from CC to TC to TT (*P* = 0.04) (Additional file 5), which however, is no longer significant after a Bonferroni correction for seven parallel tests (required *P* < 0.05/7 = 0.007). Otherwise no associations were observed between *CLU* genotypes and lipid, lipoprotein, or apolipoprotein levels.

## Discussion

The principal finding of this study is that the T allele of *CLU* rs9331896 was associated with a high risk of Alzheimer’s disease in the general population, but not with a high risk of atherosclerosis-related diseases such as vascular dementia, ischemic cerebrovascular disease, and ischemic heart disease. We used individual risk factor data, a prospective design, and the causal *APOE* rs429358 SNP (defining the ε4 allele) for adjustment to ensure the robustness of the present findings. There was no interaction between *CLU* rs9331896 and the ɛ4 allele in predicting Alzheimer’s disease risk or in predicting any of the other studied endpoints. These novel findings were observed in 103,987 individuals from the general population who were followed for up to 23 years, in 74,046 individuals from the IGAP consortium, and in 184,305 individuals from the CARDIoGRAMplusC4D consortium.

The reason for choosing clusterin, and not any of the other Alzheimer’s disease GWAS index variants is that clusterin is a biologically plausible lipid molecule with dual functions in the brain and in the peripheral circulation just like apolipoprotein E [7]. Furthermore, clusterin is the second most abundant apolipoprotein in the brain after apoE. Since the *APOE* ɛ4 allele is by far the most important genetic risk factor for Alzheimer’s disease it is plausible that a molecule resembling apoE in abundance and function is likely to play an important role in the pathogenesis of Alzheimer’s disease as well as for other endpoints. Therefore, we chose to focus on the *CLU* variant, and performed a detailed study with multiple endpoints for dementia and ischemic vascular disease.

To our knowledge, this is the first study to assess simultaneously the risk of Alzheimer’s disease, all dementia, vascular dementia, ischemic cerebrovascular disease, and ischemic heart disease as a function of a common genetic variant in *CLU* in a prospective study of the general population. A recent paper by Traylor et al. [27] identified shared genetic susceptibility between Alzheimer’s disease and small vessel disease, but not between Alzheimer’s disease and large vessel disease, thus confirming our findings for ischemic stroke. In addition to Alzheimer’s disease and ischemic stroke, we further studied vascular dementia, cerebrovascular disease including ischemic stroke, transitory ischemic attack, and amaurosis fugax, as well as ischemic heart disease and lipids and lipoproteins. Hence, the hypothesis that atherosclerosis-related diseases in general, and not only ischemic stroke, share underlying pathophysiological processes with Alzheimer’s disease could be thoroughly explored. Further, in contrast to the present study, the work by Traylor et al. [27] did not have risk factor data available to allow for adjustment for these confounding factors, and used genetic variants in partial linkage disequilibrium with ε4 instead of using the causative ε4 allele itself. The present data concludes that the major T allele of *CLU* rs9331896 is associated with a high risk of Alzheimer’s disease and all dementia, but not with atherosclerosis-related diseases like vascular dementia, ischemic cerebrovascular disease, and ischemic heart disease. Importantly, consortia data [2, 28] confirm our results with very similar risk estimates, emphasizing the strengths of the present prospective cohorts.

The exact mechanism underlying our findings is unclear. However, several lines of evidence in human and in animal models suggest plausible explanations for the present data [29–33]. In the brain, clusterin is synthesized by astrocytes and is, like apoE, bound to an HDL-like particle [7, 8]. Clusterin is a versatile protein that can serve as a chaperone for β-amyloid, and is suggested to mediate β-amyloid clearance across the blood–brain barrier via low-density lipoprotein receptor related protein 2 (LRP2, also known as megalin) [33]. Additional potential functions involve roles in apoptosis, cholesterol trafficking, inflammatory responses, and complement binding [9], and clusterin RNA brain expression is upregulated under conditions of neuronal damage [31]. Increased plasma levels of clusterin in humans have been associated with prevalent Alzheimer’s disease and with the severity of disease, but not with incident Alzheimer’s disease [34], suggesting that increased clusterin levels and/or expression may be caused by the disease, and not vice versa (in epidemiology, this phenomenon is called reverse causation). Peripherally, clusterin is, like apolipoprotein AI and paraoxonase, bound to the HDL-particle [11]. Studies in humans and mice have identified clusterin in atherosclerotic plaques of the aorta [12, 13], and clusterin levels were reported to correlate with serum paraoxonase and apolipoprotein B concentrations in Japanese men and women with coronary heart disease [35]. Interestingly, clusterin has also been shown to prevent LDL oxidation by arterial wall cells [14], suggesting that clusterin may play anti-atherogenic as well as anti-amyloidogenic roles [9], creating the rationale for investigating both atherosclerosis-related diseases and Alzheimer’s disease.

Even though our study is prospective and was performed for a large well-characterized cohort of the general population, it has potential limitations that need to be addressed. The validity of Alzheimer’s disease ICD codes was, to a certain extent, ensured by the presence of the well-known association with the ε4 allele in the present cohort, with similar risk estimates for Alzheimer’s disease as those reported globally, as well as because the core clinical criteria provide very good diagnostic accuracy and utility in most patients [36]. An intriguing finding is the trend toward a stepwise decrease in risk of ischemic heart disease associated with the T allele, which may be attributed to a higher intake of lipid-lowering therapy and possibly due to subtle increases in plasma levels of apolipoprotein B in TT and TC carriers versus CC in the population as a whole. Despite these subtle associations, the overall impression is, however, that clusterin does not play a major role in atherosclerosis-related diseases like vascular dementia and ischemic vascular disease. Finally, we studied white individuals from an ethnically homogeneous population. Consequently, our results may not necessarily apply to other ethnicities, although we are not aware of data to suggest that the present results should not apply to all ethnicities.

## Conclusions

In conclusion, a common variant in *CLU* was associated with a high risk of Alzheimer’s disease in the general population, whereas no associations with atherosclerosis-related diseases such as vascular dementia, ischemic cerebrovascular disease, and ischemic heart disease were observed. In contrast to previous studies, we used individual risk factor data, a prospective design, and the causal ε4 allele for adjustment, ensuring the robustness of the present findings. These new individual data provide independent replication of previous GWAS findings that the T allele of rs9331896 is the risk allele for Alzheimer’s disease, and we firmly conclude that *CLU* is not important for atherosclerosis- related diseases.

## Additional files

Additional file 1: Characteristics of study participants by dementia status. Characteristics of study participants with and without Alzheimer’s disease and with and without all dementia. (DOCX 17 kb)

Additional file 2: Cumulative incidences of vascular dementia and ischemic vascular disease as a function of age and rs9331896. Fine–Gray models allowing for death as a competing event were used. (PDF 188 kb)

Additional file 3: Risk of dementia and ischemic vascular disease as a function of rs9331896 in CGPS. Hazard ratios were multifactorially adjusted for age, sex, body mass index, hypertension, diabetes mellitus, smoking, alcohol consumption, physical inactivity, menopausal status and hormonal replacement therapy (only women), lipid-lowering therapy, and education (left panel). Hazard ratios were further adjusted for *APOE* genotype (middle panel). Analyses for Alzheimer’s disease, all dementia, and vascular dementia included 93,833 individuals. Analyses for ischemic cerebrovascular disease included 90,923 individuals and those for ischemic heart disease included 86,538 individuals. Analysis of individuals with the *APOE* ε33 genotype included 52,219 individuals for Alzheimer’s disease, all dementia, and vascular dementia, 50,583 for ischemic cerebrovascular disease, and 48,095 for ischemic heart disease (right panel). (PDF 17 kb)

Additional file 4: Risk of dementia and ischemic vascular disease as a function of rs9331896 in the CCHS. Hazard ratios were multifactorially adjusted for age, sex, body mass index, hypertension, diabetes mellitus, smoking, alcohol consumption, physical inactivity, menopausal status and hormonal replacement therapy (only women), lipid-lowering therapy, and education (left panel). Hazard ratios were further adjusted for *APOE* genotype (middle panel). Analyses for Alzheimer’s disease, all dementia and vascular dementia included 10,154 individuals. Analyses for ischemic cerebrovascular disease included 9971 individuals and ischemic heart disease included 9685 individuals. Analysis of individuals with *APOE* ε33 genotype included 5704 individuals for Alzheimer’s disease, all dementia and vascular dementia, 5601 for ischemic cerebrovascular disease and 5443 for ischemic heart disease (right panel). (PDF 18 kb)

Additional file 5: Lipid, lipoprotein, and apolipoprotein levels as a function of rs9331896. Values are mean ± standard error of the mean (SEM) for total cholesterol, LDL cholesterol, apolipoprotein B, HDL cholesterol, and apolipoprotein AI levels, and geometric mean ± SEM for apolipoprotein E and triglyceride levels. To convert cholesterol values to mg/dL, divide them by 0.0259 and to convert triglyceride values to mg/dL, divide them by 0.0113. HDL high-density lipoprotein, LDL low-density lipoprotein. (PDF 10 kb)

## Abbreviations

*ADGC*: Alzheimer’s Disease Genetics Consortium
*APOE*: *apolipoprotein E* (gene)
apoE: apolipoprotein E (protein)
*APOJ*: *apolipoprotein J (*gene)
apoJ: apolipoprotein J (protein)
CARDIoGRAMplusC4D: the Coronary Artery Disease Genome-wide Replication and Meta-analysis plus the Coronary Artery Disease (C4D) Genetics and 1000-Genomes-based GWAS
CCHS: Copenhagen City Heart Study
CGPS: Copenhagen General Population Study
CHARGE: Cohorts for Heart and Aging Research in Genomic Epidemiology Consortium
CI: Confidence interval
*CLU*: *clusterin (gene)*
EADI: European Alzheimer’s Disease Initiative
f: frequency
GERAD: Genetic and Environmental Risk in Alzheimer’s Disease Consortium
GWAS: Genome-wide association study
HDL: high-density lipoprotein
ICD: International Classification of Diseases
IGAP: International Genomics of Alzheimer’s Project
LDL: low-density lipoprotein
LRP2: low-density lipoprotein receptor related protein 2
NIH: National Institutes of Health
RNA: ribonucleic acid
SNP: single-nucleotide polymorphism
WHO: World Health Organization
