# Supplementary material for: Genetic variation in clusterin and risk of dementia and ischemic vascular disease in the general population: cohort studies and meta-analyses of 362,338 individuals
Source: BMC Med. 2018 Mar 14;16:39. doi: 10.1186/s12916-018-1029-3 (PMC5851250; doi:10.1186/s12916-018-1029-3)
Supplement: Supplementary file 1 — Characteristics of study participants by dementia status. Characteristics of study participants with and without Alzheimer’s disease and with and without all dementia. (DOCX 17 kb) [file 12916_2018_1029_MOESM1_ESM.docx]

**Additional file 1**. Characteristics of study participants by disease status

|  | Without Alzheimer disease | With  Alzheimer disease | P | Without all dementia | With all dementia | P |
| --- | --- | --- | --- | --- | --- | --- |
| No. of individuals (%) | 103,095 (99.3) | 892 (0.9) |  | 102,098 (98.4) | 1,889 (1.8) |  |
| Age (years) | 58(48-67) | 73(67-78) | 2*10^-231^ | 58(48-67) | 73(67-79) | <1*10^-300^ |
| Female (%) | 55 | 62 | 5*10^-4^ | 55 | 59 | 1*10^-2^ |
| Total cholesterol (mmol/L) | 5.6( 4.9- 6.3) | 6.0( 5.2- 6.8) | 4*10^-24^ | 5.6(4.9-6.3) | 6(5.2-6.8) | 3*10^-42^ |
| LDL cholesterol (mmol/L) | 3.2( 2.6- 3.9) | 3.5 (2.8- 4.3) | 8*10^-14^ | 3.2(2.6-3.9) | 3.5(2.8-4.2) | 3*10^-23^ |
| HDL cholesterol  (mmol/L) | 1.6(1.2-1.9) | 1.7( 1.4-2.0) | 6*10^-10^ | 1.6(1.2-1.9) | 1.6(1.3-2.4) | 4*10^-8^ |
| Triglycerides  (mmol/L) | 1.4 ( 1.0-2.1) | 1.5 (1.1-2.1) | 4*10^-3^ | 1.4(1.0-2.1) | 1.5(1.1-2.2) | 9*10^-13^ |
| Body mass index (kg/m^2^) | 26 (23-28) | 25 (23-28) | 1*10^-2^ | 26(23-28) | 26(23-28) | 0.7 |
| Hypertension (%) | 59 | 78 | 3*10^-29^ | 60 | 59 | 3*10^-53^ |
| Diabetes mellitus (%) | 4 | 5 | 4*10^-2^ | 4 | 7 | 7*10^-14^ |
| Smoking (%) | 21 | 24 | 4*10^-2^ | 21 | 29 | 1*10^-17^ |
| Alcohol consumption (%) | 17 | 16 | 2*10^-1^ | 17 | 15 | 2*10^-2^ |
| Physical inactivity (%) | 51 | 59 | 4*10^-7^ | 50 | 65* | 9*10^-40^ |
| Postmenopausal (%)^a^ | 67 | 98 | 1*10^-62^ | 66 | 98 | 1*10^-121^ |
| Hormonal replacement therapy (%)^a^ | 11 | 16 | 1*10^-61^ | 11 | 15* | 1*10^-120^ |
| Lipid-lowering therapy (%) | 11 | 13 | 8*10^-2^ | 11 | 13 | 1*10^-2^ |
| Education < 8 years | 12 | 37 | 3*10^-112^ | 12 | 39 | 8*10^-278^ |

Values are median (interquartile range) or percent, and are from the day of enrollment in 2003 and onwards for CGPS and 1991-1994 or 2001-2003 and onwards for CCHS. Missing values (0.7%) were imputed (continuous covariates) or assigned a dummy value (categorical covariates). Hypertension was use of anti-hypertensive medication and/or a systolic blood pressure of 140 mm Hg or greater, and/or a diastolic blood pressure of 90 mm Hg or greater. Diabetes mellitus was self-reported disease, use of insulin or oral hypoglycaemic agents, and/or non-fasting plasma glucose levels of more than 11 mmol/L (>198 mg/dL). Smoking was current smoking. Alcohol consumption was >14/21 units per week for women/men (1 unit=12 g alcohol, equivalent to one glass of wine or one beer (33 cL)). Physical inactivity was ≤four hours per week of light physical activity in leisure time. Women reported menopausal status and use of hormonal replacement therapy. Lipid- lowering therapy was primarily statins (yes/no), and education was <8 years of education. ^a^In women only.
