# Supplementary figures and images for: Genetic variation in clusterin and risk of dementia and ischemic vascular disease in the general population: cohort studies and meta-analyses of 362,338 individuals
Source: BMC Med. 2018 Mar 14;16:39. doi: 10.1186/s12916-018-1029-3 (PMC5851250; doi:10.1186/s12916-018-1029-3)

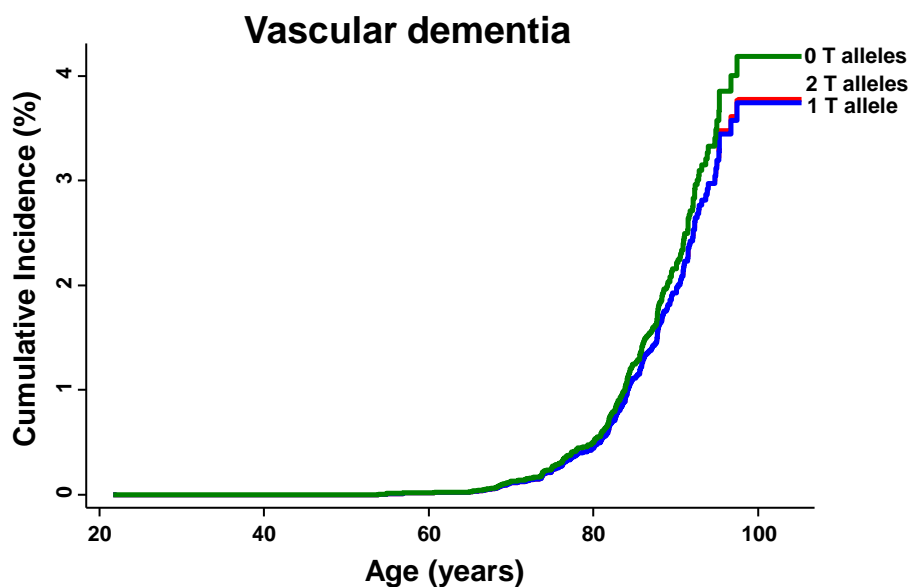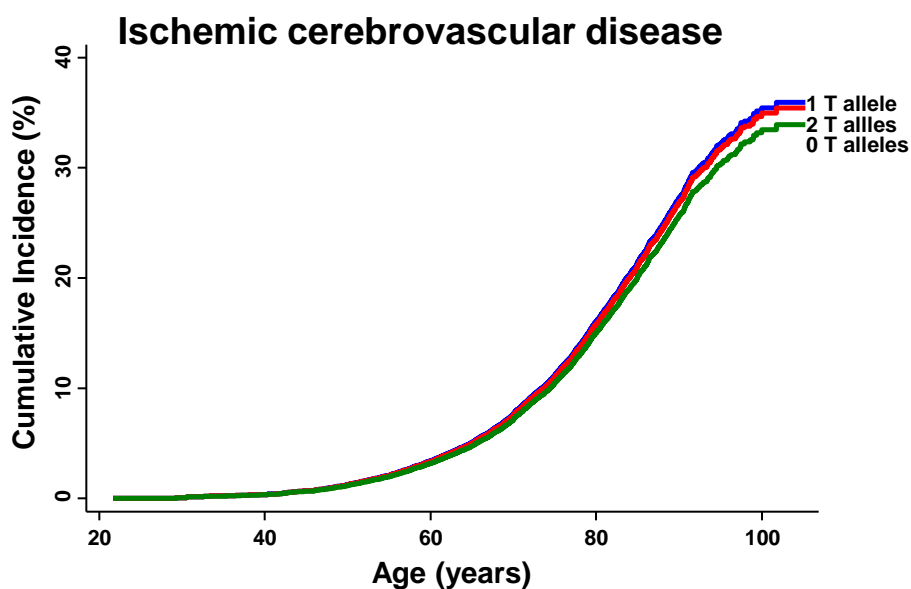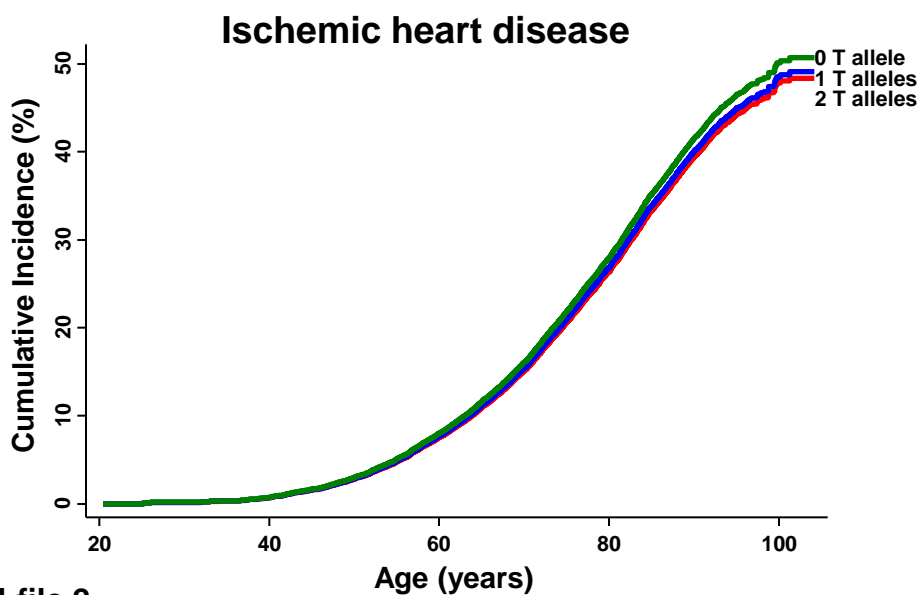

Supplement: Supplementary file 2 — Cumulative incidences of vascular dementia and ischemic vascular disease as a function of age and rs9331896. Fine–Gray models allowing for death as a competing event were used. (PDF 188 kb) [file 12916_2018_1029_MOESM2_ESM.pdf]

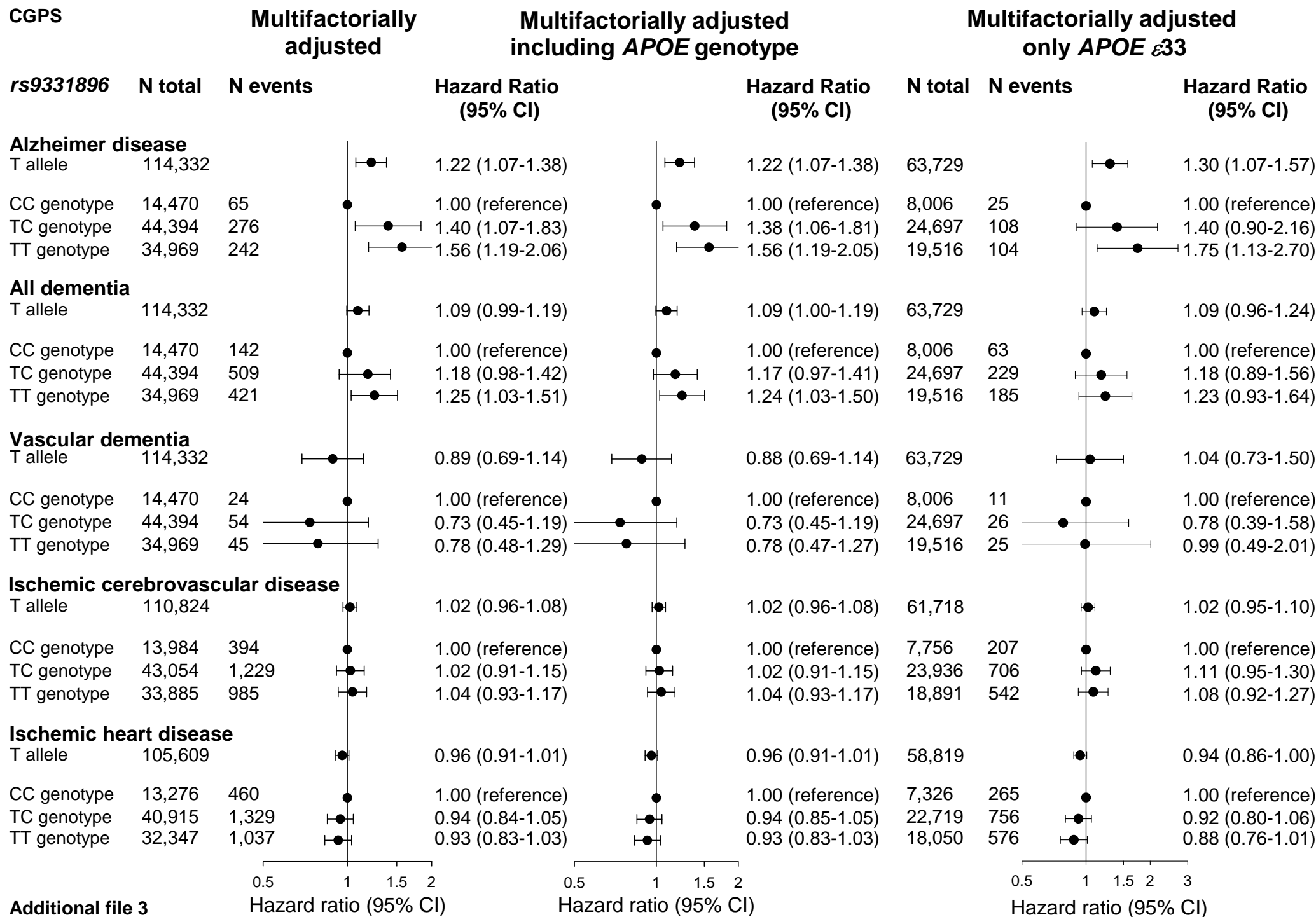

Supplement: Supplementary file 3 — Risk of dementia and ischemic vascular disease as a function of rs9331896 in CGPS. Hazard ratios were multifactorially adjusted for age, sex, body mass index, hypertension, diabetes mellitus, smoking, alcohol consumption, physical inactivity, menopausal status and hormonal replacement therapy (only women), lipid-lowering therapy, and education (left panel). Hazard ratios were further adjusted for APOE genotype (middle panel). Analyses for Alzheimer’s disease, all dementia, and vascular dementia included 93,833 individuals. Analyses for ischemic cerebrovascular disease included 90,923 individuals and those for ischemic heart disease included 86,538 individuals. Analysis of individuals with the APOE ε33 genotype included 52,219 individuals for Alzheimer’s disease, all dementia, and vascular dementia, 50,583 for ischemic cerebrovascular disease, and 48,095 for ischemic heart disease (right panel). (PDF 17 kb) [file 12916_2018_1029_MOESM3_ESM.pdf]

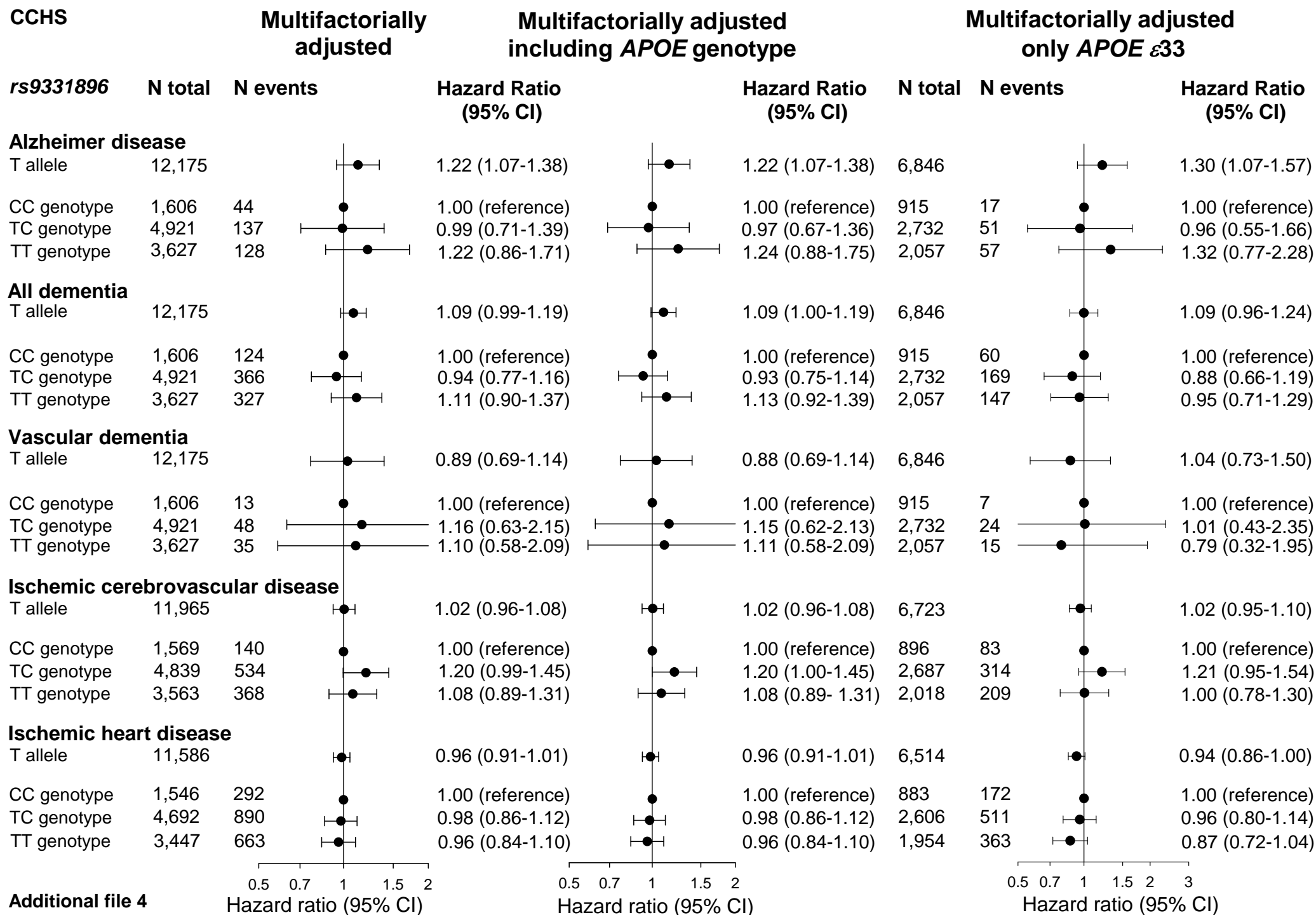

Supplement: Supplementary file 4 — Risk of dementia and ischemic vascular disease as a function of rs9331896 in the CCHS. Hazard ratios were multifactorially adjusted for age, sex, body mass index, hypertension, diabetes mellitus, smoking, alcohol consumption, physical inactivity, menopausal status and hormonal replacement therapy (only women), lipid-lowering therapy, and education (left panel). Hazard ratios were further adjusted for APOE genotype (middle panel). Analyses for Alzheimer’s disease, all dementia and vascular dementia included 10,154 individuals. Analyses for ischemic cerebrovascular disease included 9971 individuals and ischemic heart disease included 9685 individuals. Analysis of individuals with APOE ε33 genotype included 5704 individuals for Alzheimer’s disease, all dementia and vascular dementia, 5601 for ischemic cerebrovascular disease and 5443 for ischemic heart disease (right panel). (PDF 18 kb) [file 12916_2018_1029_MOESM4_ESM.pdf]

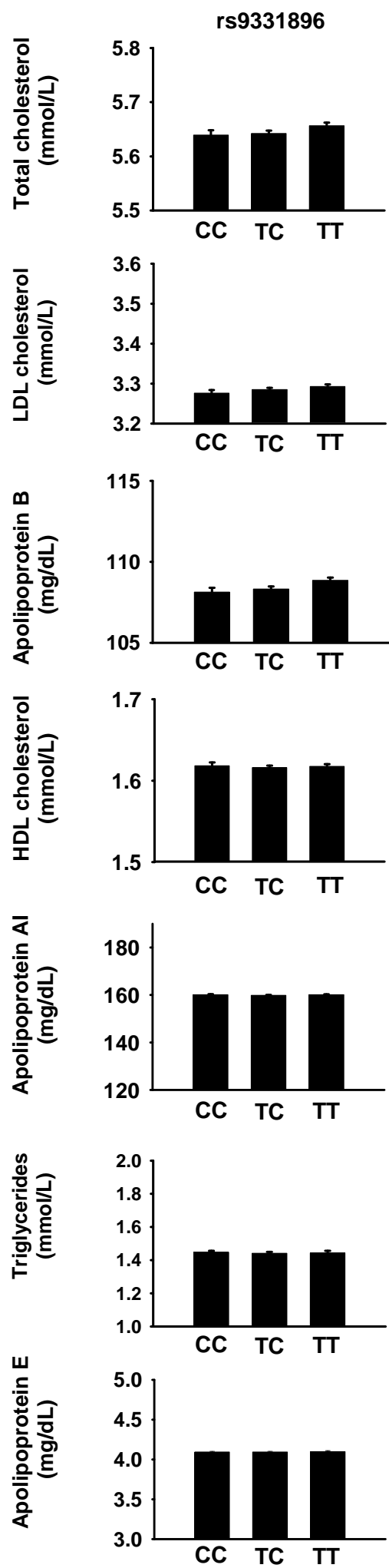

16,076 49,315 38,596

Supplement: Supplementary file 5 — Lipid, lipoprotein, and apolipoprotein levels as a function of rs9331896. Values are mean ± standard error of the mean (SEM) for total cholesterol, LDL cholesterol, apolipoprotein B, HDL cholesterol, and apolipoprotein AI levels, and geometric mean ± SEM for apolipoprotein E and triglyceride levels. To convert cholesterol values to mg/dL, divide them by 0.0259 and to convert triglyceride values to mg/dL, divide them by 0.0113. HDL high-density lipoprotein, LDL low-density lipoprotein. (PDF 10 kb) [file 12916_2018_1029_MOESM5_ESM.pdf]
